# Reward Learning as a Potential Mechanism for Improvement in Schizophrenia Spectrum Disorders Following Cognitive Remediation: Protocol for a Clinical, Nonrandomized, Pre-Post Pilot Study

**DOI:** 10.2196/52505

**Published:** 2024-01-22

**Authors:** Frances Dark, Graham Galloway, Marcus Gray, Matteo Cella, Veronica De Monte, Victoria Gore-Jones, Gabrielle Ritchie

**Affiliations:** 1 Metro South Addiction and Mental Health Services Woolloongabba Australia; 2 Faculty of Medicine University of Queensland Brisbane Australia; 3 Translational Research Institute Woolloongabba Australia; 4 Herston Imaging Research Facility The University of Queensland Brisbane Australia; 5 King's College London London United Kingdom

**Keywords:** cognitive remediation, fMRI, functional magnetic resonance imaging, negative symptoms, psychosocial functioning, reward learning

## Abstract

**Background:**

Cognitive impairment is common with schizophrenia spectrum disorders. Cognitive remediation (CR) is effective in improving global cognition, but not all individuals benefit from this type of intervention. A better understanding of the potential mechanism of action of CR is needed. One proposed mechanism is reward learning (RL), the cognitive processes responsible for adapting behavior following positive or negative feedback. It is proposed that the structure of CR enhances RL and motivation to engage in increasingly challenging tasks, and this is a potential mechanism by which CR improves cognitive functioning in schizophrenia.

**Objective:**

Our primary objective is to examine reward processing in individuals with schizophrenia before and after completing CR and to compare this with a group of matched clinical controls. We will assess whether RL mediates the relationship between CR and improved cognitive function and reduced negative symptoms. Potential differences in social RL and nonsocial RL in individuals with schizophrenia will also be investigated and compared with a healthy matched control group.

**Methods:**

We propose a clinical, nonrandomized, pre-post pilot study comparing the impact of CR on RL and neurocognitive outcomes. The study will use a combination of objective and subjective measures to assess neurocognitive, psychiatric symptoms, and neurophysiological domains. A total of 40 individuals with schizophrenia spectrum disorders (aged 18-35 years) will receive 12 weeks of CR therapy (n=20) or treatment as usual (n=20). Reward processing will be evaluated using a reinforcement learning task with 2 conditions (social reward vs nonsocial reward) at baseline and the 12-week follow-up. Functional magnetic resonance imaging responses will be measured during this task. To validate the reinforcement learning task, RL will also be assessed in 20 healthy controls, matched for age, sex, and premorbid functioning. Mixed-factorial ANOVAs will be conducted to evaluate treatment group differences. For the functional magnetic resonance imaging analysis, computational modeling will allow the estimation of learning parameters at each point in time, during each task condition, for each participant. We will use a variational Bayesian framework to measure how learning occurred during the experimental task and the subprocesses that underlie this learning. Second-level group analyses will examine how learning in patients differs from that observed in control participants and how CR alters learning efficiency and the underlying neural activity.

**Results:**

As of September 2023, this study has enrolled 15 participants in the CR group, 1 participant in the treatment-as-usual group, and 11 participants in the healthy control group. Recruitment is expected to be completed by September 2024. Data analysis is expected to be completed and published in early 2025.

**Conclusions:**

The results of this study will contribute to the knowledge of CR and RL processes in severe mental illness and the understanding of the systems that impact negative symptoms and cognitive impairments within this population.

**International Registered Report Identifier (IRRID):**

DERR1-10.2196/52505

## Introduction

### Overview

Schizophrenia spectrum disorders (SSDs; eg, schizophrenia, schizoaffective disorder, and schizophreniform disorder) are defined by the presentation of a range of symptoms, including delusions, hallucinations, disorganized thoughts and behaviors, and negative symptoms (ie, reduced motivation and emotional expression) [1]. These disorders are linked to a variety of deficits in cognition, which extend to both neurocognition (eg, attention, memory, and planning) and social cognition (eg, difficulties perceiving and processing emotions) [2]. Both stand-alone and integrated programs have been used to treat these cognitive impairments. Cognitive remediation (CR) therapy has been widely used as an intervention for deficits in global cognition and functional difficulties in schizophrenia and has been shown to be particularly beneficial if rehabilitation is also incorporated into treatment [3,4]. Nevertheless, response to therapy is variable [5], and the impact individual characteristics have on the success of CR is still being investigated. The mechanisms by which CR is effective are yet to be clarified. A core component of CR is strategic learning principles, which ensure tasks are scaffolded based on previous successful achievements and the chances of successful task completion are optimized. There is, therefore, close reinforcement of learning.

One proposed mechanism for the effect of CR is reward learning (RL), which is potentially the pathway to improved cognition and motivational negative symptoms. In schizophrenia, there is impairment in reward anticipation [6,7] and representation [8], leading to poorer decision-making and goal-directed behavior, motivational deficits, and negative symptoms [8-11]. RL is a term used to identify the cognitive processes responsible for adapting behavior following positive or negative feedback. RL is a basic adaptive function of every living organism and provides the possibility to adapt and change in response to internal and environmental demands [12]. This process has been extensively studied in neuroscience and linked to the brain dopamine system. The dopamine hypothesis of schizophrenia is the single most influential theory in our understanding of the neurochemical basis of the disorder [13]. This theory suggests that fundamental dysregulation in this system is responsible for the illness’s symptoms. Dysregulation in the dopamine system is also linked to RL abnormalities, which, in turn, are thought to influence cognitive and negative symptoms. A growing body of basic neuroscience literature has identified 2 complementary and interactive neural systems in the dopamine system responsible for predicting outcomes and learning from feedback [14]. The first of these systems, responsible for rapid learning, is mediated by the basal ganglia. This system, referred to as the “fast system,” is believed to represent the predicted value of actions and rewards. These predictions bias actions and underlie learning based on positive and negative feedback. The second slower system is based primarily in the prefrontal cortex and allows for more detailed, conscious, and abstract representations of values and rewards. These representations of value are instrumental in allowing individuals to flexibly respond to reward value and adapt to novelty in the environment.

There is consistent evidence that people with SSDs are impaired at making rapid behavioral adjustments in response to feedback and that these impairments are associated with negative and cognitive symptoms [15-17]. Problems using the “fast system” are evident in situations requiring rapid change in responses to environmental changes when a situation previously rewarding begins to be associated with disadvantageous outcomes and oversensitivity to negative feedback and poor sensitivity to positive feedback. In contrast, several studies suggest that the gradual or procedural learning system seems intact in people with schizophrenia [18], but antipsychotic medication dosage, particularly for those with high levels of dopamine 2 receptor blockades, may exert a negative effect on this system.

Social environments are dynamic with constant rapid changes; hence, social situations require rapid behavioral adjustments in response to ever-changing social feedback. People with SSDs have impaired social functioning, and recent studies have shown that they also have impaired social reward processing [19,20]. Social approval induces rewarding feelings and is associated with increased activation in regions and networks associated with reward [21-23]. In those with SSDs, there is reduced activity in common reward brain regions during the experience of social reward [24], suggesting that they may have a reduced experience of the rewarding feeling of positive social attention. Positive social interactions have benefits for mental well-being and give life a sense of meaning [25]. Receiving praise and attention from others improves self-esteem [26] and increases motivation [27]. Although social reward has major impacts on functional outcomes, only recent efforts have explored social reward processing in SSDs. Further behavioral evidence suggests that RL difficulties are more pronounced in learning from positive, rather than negative, feedback [8]. This provides a further link between the effects of impaired RL and negative symptoms. Learning preferentially from negative outcomes is likely to lead to behavioral avoidance and social withdrawal, and have a negative impact on motivation. This hypothesis is supported by research suggesting that the magnitude of RL impairment, particularly for positive feedback, is associated with negative symptom severity [28].

Despite the significance of RL problems in people with SSDs, there is no therapy targeting this problem. One previous study explored the impact that a course of CR has on RL problems in people with schizophrenia [29]. The results of this study showed that CR could improve sensitivity to positive and negative feedback and that improvement in these parameters was moderated by the severity of negative symptoms. However, this study used a standard CR protocol and may not have achieved the maximum effect on RL problems. Furthermore, the nature of this study did not allow for investigating the retention of RL improvements and, more crucially, how these may impact cognitive and negative symptoms and, more broadly, recovery. RL difficulties in people with SSDs are associated with negative symptoms, and it is plausible that, by reducing RL difficulties, a reduction in negative symptoms could be observed. It is proposed that the structure of CR enhances rewards and motivation to engage in increasingly challenging tasks, and this is a potential mechanism by which CR can achieve functional outcomes in individuals with SSDs.

### Aims and Hypotheses

Our primary aim is to investigate reward processing in individuals with SSDs before and after completing a course of CR and to compare this intervention group with a treatment-as-usual (TAU) group of individuals with SSDs. In addition, this study aims to investigate whether RL mediates changes in cognitive function and negative symptoms following CR. We will also examine potential differences in social RL compared with nonsocial RL in individuals with SSDs and the impact of CR on these potential differences in RL domains. Comparison with RL in a healthy adult control group will allow further differentiation of behavioral and neural impairments in SSDs.

#### Hypothesis 1A

At baseline, participants with SSDs will demonstrate deficits in RL when compared with healthy control volunteers. These differences in learning will be linked to aberrant activity in the dopamine system at a neural level in the prefrontal cortex and subcortical structures such as the basal ganglia, compared with healthy controls.

#### Hypothesis 1B

At baseline, participants with SSDs will demonstrate greater deficits in social RL when compared with nonsocial RL.

#### Hypothesis 2

Participants that complete CR will demonstrate improved RL, again reflecting improved neural activity within the prefrontal and basal ganglia regions, when compared with people with SSDs not receiving the intervention.

#### Hypothesis 3

Reward processing ability will mediate improved cognition following CR in participants receiving the intervention but not in the SSD control group.

#### Hypothesis 4

Reward processing will mediate improvements in negative symptoms following CR in participants receiving the intervention but not in the SSD control group.

## Methods

### Study Design

**Figure 1:**
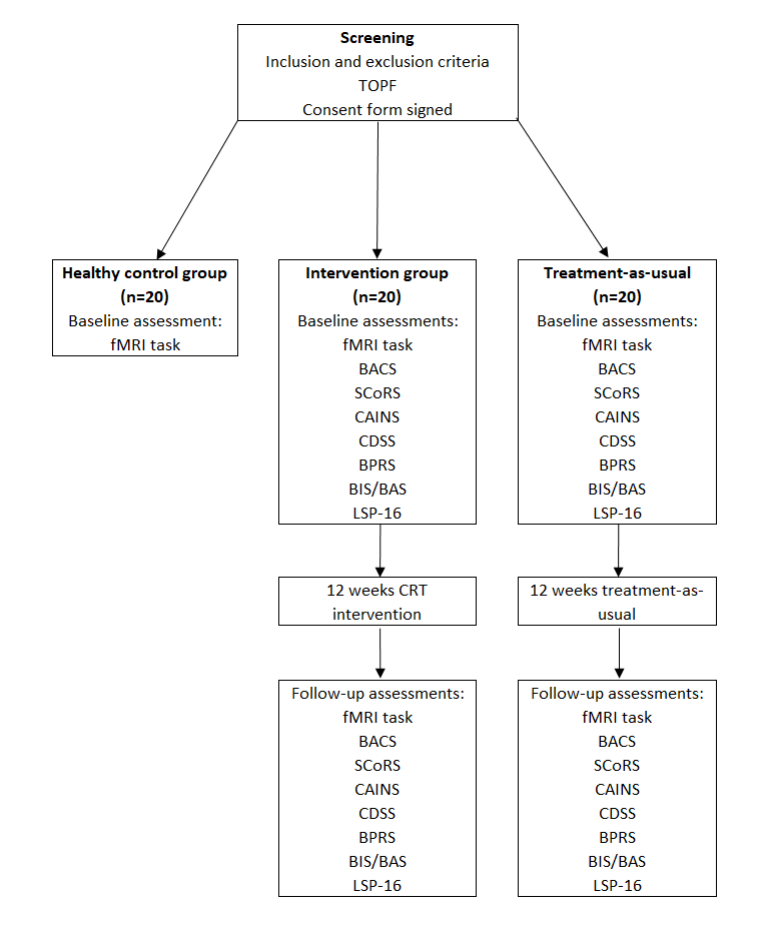
The reward learning as a potential mechanism for improvement in schizophrenia spectrum disorders following cognitive remediation pre-post pilot study design. BACS: Brief Assessment of Cognition in Schizophrenia; BIS/BAS: Behavioral Inhibition System and Behavioral Activation System; BPRS: Brief Psychiatric Rating Scale; CAINS: Clinical Assessment Interview for Negative Symptoms; CDSS: Calgary Depression Scale for Schizophrenia; CRT: cognitive remediation therapy; fMRI: functional magnetic resonance imaging; LSP-16: Life Skills Profile; SCoRS: Schizophrenia Cognition Rating Scale; TOPF: Test of Premorbid Functioning.

The study is a nonrandomized clinical pilot trial to investigate whether reward processing pathways are involved in the mechanism of action of cognitive remediation therapy (Computerized Interactive Remediation of Cognition-Training for Schizophrenia [CIRCuiTS]) [30,31]. We will recruit 3 participant groups: 20 participants with SSDs who will complete the CR program (the intervention group), 20 participants with SSDs who will receive TAU, and 20 matched healthy control participants. Figure 1 depicts a schematic of the study design.

### Participants

A total of 40 participants with a diagnosis of an SSD will be recruited from the Metro South Addiction and Mental Health Service community teams (Brisbane, Australia). Given that this is a pilot study, a power analysis was not conducted [32]. Instead, the sample size was informed by practical factors relating to the project, including the budget, the availability of CR facilitators, and the recruitment and attrition rates from our previous studies. This sample size is also consistent with other research in this area [33].

The inclusion criteria for the intervention and TAU groups and the healthy control group, as well as the exclusion criteria for all groups, have been provided in Textbox 1.

**The inclusion and exclusion criteria.**

**Inclusion criteria for the intervention and treatment-as-usual (TAU) groups**

- Patients of the Metro South Addiction and Mental Health Service
- Aged between 18 and 35 years
- Primary diagnosis of schizophrenia spectrum disorder (SSD)
- English literacy skills of at least grade 4 equivalence
- Absence of neurological disorders or acquired brain injury
- Estimated intelligence quotient >70 on the Test of Premorbid Functioning (TOPF)
- Agree to participate and have the capacity to consent to the study procedures

**Inclusion criteria for the healthy control group**

- Aged between 18 and 35 years
- No history of diagnosis of SSD
- English literacy skills of at least grade 4 equivalence
- Absence of neurological disorders or acquired brain injury
- Estimated intelligence quotient >70 on the TOPF
- Agree to participate and have the capacity to consent to the study procedures

**Exclusion criteria for all groups**

- Metallic object in their body (eg, cardiac pacemaker and cochlear implant)
- Pregnant or possibly pregnant (unprotected sex since last menstrual period)
- History of claustrophobia
- Permanent metal dental appliances
- Bodyweight ≥120kg

The researchers will obtain consent from all participants through a participation information and consent form. For participants with SSDs, half (n=20) will complete CR and form the intervention group. Those participants with SSDs (n=20) that are not interested in completing CR will form the patient TAU group and will complete the pre- and postmeasures only. Thus, group allocation will be based on an individual’s preference to participate in CR. In addition, matched healthy controls (n=20) will be recruited from the Metro South Addiction and Mental Health community services in the Princess Alexandra Hospital district as well as from the general population. We do not anticipate that it will be difficult to collect these healthy controls, given our already established connections with other researchers and staff within this district. Therefore, we expect to be able to recruit this group through word of mouth and the snowball effect. These health controls will have no history of SSDs. This will provide a benchmark to compare the clinical groups. Hence, the total number of participants for the study is 60.

### Intervention

CIRCuiTS is a therapist-supported CR web-based program that focuses on improving cognitive skills, particularly for individuals with psychosis [30,31]. Participants work through different cognitive tasks and exercises, many of which are based on real-life experiences (eg, creating a shopping list or cooking). Previous studies have shown CIRCuiTS leads to improvements in both cognition and functional recovery and is acceptable by participants [34]. Sessions typically last 1 hour, twice a week, for 12 weeks, and task practice is delivered through a computer. CIRCuiTS consists of 40 stages. A total of 20 sessions are considered adequate treatment exposure, and 20 minutes is the minimum time for a “session.” In this study, participants in the CR group will have 1 face-to-face meeting with the therapist to orient themselves to the program. These participants will then complete the program either on the web or in person, in a group or individually. Only the intervention group will be able to complete these sessions.

All participants will continue their usual treatment under the supervision of their referring clinical team. This involves pharmacotherapy, monitoring, and case management. They can concurrently attend any psychosocial group that does not focus on improving neurocognition.

### Outcomes

#### Screening Measures

During screening, participants will complete the Test of Premorbid Functioning (TOPF). The TOPF is a test of premorbid intelligence estimated from reading ability and will be used to screen for intellectual impairment [35,36]. It takes approximately 10 minutes to complete and is composed of a list of 70 words.

#### Demographic Information

At baseline, demographic and clinical information will be gathered, including sex, age, and date of birth. For the clinical groups, the case management team, primary and secondary diagnoses, mental health status, and list of current medications (name, dose, frequency, and route) will also be recorded.

A battery of validated assessment measures will also be delivered at baseline and follow-up for the clinical groups, as described in the subsequent sections.

#### Brief Assessment of Cognition in Schizophrenia

The Brief Assessment of Cognition in Schizophrenia (BACS) assesses 5 domains of cognition, with 6 tests taking approximately 30 minutes [37,38]. The 6 tests include list learning (verbal memory), digit sequencing (working memory), token motor task (motor speed), verbal fluency (semantic fluency and letter fluency), Tower of London (reasoning and problem solving), and symbol coding (attention and processing speed). The BACS has high test-retest reliability, is sensitive to the unique cognitive deficits associated with SSDs, and is a routine measure of change in performance over time [37].

#### Schizophrenia Cognition Rating Scale

The Schizophrenia Cognition Rating Scale (SCoRS) is a 20-item measure of cognitive difficulties in daily activities that is completed by the participant, an informant, and the interviewer at baseline and on completion of the CR program [39]. It has been found that global ratings of cognition are strongly correlated with cognitive performance, functional outcome, and functional capacity. The SCoRS has good interrater reliability [39].

#### Clinical Assessment Interview for Negative Symptoms

The Clinical Assessment Interview for Negative Symptoms (CAINS) is a measure of anhedonia, avolition, and emotional expression [40] with strong psychometric properties [41]. The CAINS has been found to have good convergent validity with the Brief Negative Symptom Scale [42]. It was developed to better align not only with the negative symptoms but also with constructs emerging from neurobiological research [43].

#### Calgary Depression Scale for Schizophrenia

The Calgary Depression Scale for Schizophrenia (CDSS) measures symptoms of depression in people with schizophrenia [44]. The scale has 8 structured questions with an additional observational item [45]. The scoring uses a 4-point Likert-type scale (0=absent, 1=mild, 2=moderate, and 3=severe), anchored by descriptors [44]. The summation of scores on all items provides a global score. The scale has good psychometric properties, identifying a major depressive episode at 82% specificity and 85% sensitivity for scores above 6.

#### Brief Psychiatric Rating Scale

The Brief Psychiatric Rating Scale (BPRS) is a semistructured assessment of 24 symptoms of schizophrenia. The 24-item anchored scale will be used. The anchored version has good psychometric properties [46]. Symptom severity is rated from 1 (not present) to 7 (extremely severe). High scores represent greater symptom severity. Based on a clinical interview, items 1-14 are based on the participants’ self-report; observed behavior is also used to rate items 7, 12, and 13. Items 15-24 are rated based on the patient’s observed behavior or speech during the interview.

#### Behavioral Inhibition System and Behavioral Activation System

The Behavioral Inhibition System and Behavioral Activation System (BIS/BAS) is a 24-item self-report measure of behavioral inhibition and activation [46,47]. A total of 13 items reflect the activation system, divided into drive, pleasure-seeking, and sensitivity to reward, and 7 items reflect the inhibition system. There are 4 filler items. Responses are rated on a 4-point Likert-type scale with a range from 1 (very true) to 4 (very false). The BIS/BAS factor structure has been validated in a large Australian community sample [48].

#### Life Skills Profile

The Life Skills Profile (LSP-16) is a measure of activities of daily living over the previous 3 months with high test-retest and interrater reliability [49]. The 16-item version was specifically developed for use in Australian public mental health services [49-51]. The items are on a 4-point anchored scale. Higher scores indicate a greater disability. A total LSP score is calculated by adding all the individual scores.

#### Functional Magnetic Resonance Imaging

**Figure 2:**
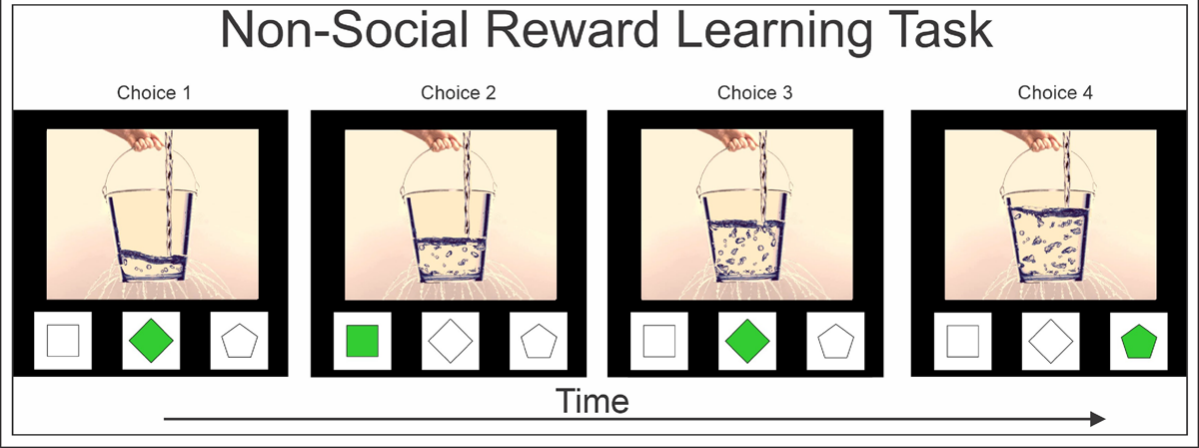
The experimental learning task with the nonsocial reward condition in the pre-post pilot study. Choice 4 represents the best outcome.

Participants will complete a computerized experimental learning task with 2 conditions: a social reward condition and a nonsocial reward condition (Figure 2). The task is a simple reinforcement learning design where participants must choose from a small number of potential responses and, over repeated trials, learn about the probable consequences. In our task, participants can choose a response from 3 buttons. Each choice is associated with a probabilistic outcome, generally with 1 winning choice, 1 losing choice, and 1 neutral choice. For example, the task might start with an 80% reward for choice A, a 40% loss for choice B, and no change for choice C. With repeated sampling, people learn to identify responses with the best outcomes and avoid irrelevant or losing responses.

During the social learning task, participants view a picture of a neutral face, with rewarded button choices changing the facial expression toward a smile and losing choices changing toward a frowning expression. Alternatively, in the nonsocial learning task, participants view a picture of a bucket half full of water. Rewarded choices further fill the bucket, while losing choices drain the bucket. The task was developed by Dr Marcus Gray for this study and implemented in MATLAB (MathWorks) using the Cogent 2000 toolbox [52]. During the functional magnetic resonance imaging (fMRI) experiment, the task is seen by participants through a tilted mirror attached to the head coil on the magnetic resonance imaging scanner. Responses are made on a commercially available, magnetic resonance-compatible response box [53]. Participants are familiarized with the task and perform 2 blocks of each condition before brain scanning.

During the fMRI, structural and functional magnetic resonance imaging images will also be acquired by a 3T Siemens Magnetom TrioTim (Siemens Healthineers) system using a 12-channel head coil. The sequences acquired and their parameters are as follows: T1-weighted imaging, T2-weighted imaging, fMRI imaging, diffusion-weighted imaging, and susceptibility-weighted imaging.

- T1-weighted imaging: magnetization-prepared 2 rapid acquisition gradient echoes (MP2RAGE) sequence. Time to acquire image=5:02; inversion 1=700 ms; inversion 2=2220 ms; repetition time=4000 ms; echo time=2.96 ms; voxel size=1 mm isotropic; field of vision=230 mm; and 192 slices with full brain coverage.
- T2-weighted imaging: fluid-attenuated inversion recovery sequence. Time to acquire image=2:44; repetition time=9000 ms; echo time=81 ms; inversion time=2500 ms; flip angle=150 degrees; voxel size = 0.72 × 0.72 × 5.2 mm; and 30 slices with full brain coverage.
- fMRI imaging: functional T2*-weighted blood-oxygen-level dependent images are acquired using a multiband, echo-planar sequence across the whole brain (repetition time=0.628 ms; echo time=30 ms; resolution=2.4 mm isotropic; field of view=192 mm; flip angle=52 degrees; and 54 slices with full brain coverage). During fMRI imaging, participants will complete a computerized experimental task. For each task condition, approximately 720 full brain images will be acquired, providing 1440 volumes acquitted in approximately 12 minutes.
- Diffusion-weighted imaging: A neurite orientation dispersion and density imaging sequence with 2 shells and 90 gradient directions (B1=1000 with 30 directions and B2=2500 with 60 directions) with 6 B0 measurements will be acquired in the anterior-posterior phase encoding direction, and an additional 6 B0 measurements will be acquired in the posterior-anterior phase encoding direction. Total acquisition time=7:24; repetition time=4100 ms; echo time=75 ms; voxel size=2mm isotropic; and 68 slices with full brain coverage.
- Susceptibility-weighted imaging: time to acquire image=2:56; repetition time=27 ms; echo time=20 ms; flip angle=15 degrees; voxel size = 0.89 × 0.89 × 2.5 mm; and 64 slices with full brain coverage.

### Data Management

The Trial Management Group (TMG) consists of the principal investigator (FD) and associated investigators (GG and MG). All adverse events will be reviewed by the TMG and reported to the ethics committee. The process of recruitment and data management will be overseen by the TMG. The data will be securely entered and stored on the University of Queensland Data Manager repository. This trial may be subject to random auditing by the ethics committee. All protocol amendments will be reported to the ethics committee.

### Statistical Analysis

Participants’ medication dosages will be converted to olanzapine equivalents [54]. Outcome measures will be analyzed using the SPSS (version 27 or higher; IBM Corp) software package. A series of 2 (group: intervention and patient control) x 2 (time: baseline and postintervention) mixed factorial ANOVAs will be conducted to evaluate the treatment group differences for each of the outcome measures. If the normality assumption is violated, nonparametric analyses will be conducted.

For the fMRI analysis, standard preprocessing of the functionally weighted images will be carried out using the Statistical Parametric Mapping Version 12 [55]. The preprocessing steps follows: slice timing on the functional images, to correct for differences in slice acquisition times within each volume using the middle slice as reference; realignment (estimate and reslice) on the functional images, to correct for interscan movement within each run (defined as >3 mm translation and >2 degrees rotation); coregistration of the functional and structural images; segmentation of the structural image, with heavy regularization (0.1) recommended for MP2-RAGE sequence; normalization of the resliced images into a standardized, stereotaxic space (according to the Montreal Neurological Institute template); and smoothing of normalized images with a 8 mm full-width-at-half-maximum isotropic Gaussian kernel.

The general linear model approach for event-related designs will be conducted using the Statistical Parametric Mapping Version 8 [55]. For the first-level analysis, task-related changes in the blood-oxygen-level dependent signal will be estimated at each voxel for each participant. Head motion parameters will be included as a regressor to account for participant motion during the experiment. A 1/128 Hz high-pass filter will be used to remove slow signal drifts, and a canonical hemodynamic response function with no derivatives will be selected.

Computational modeling will allow the estimation of learning parameters at each point in time, during each task condition, for each participant. We will use a variational Bayesian framework to compute how the value of each button was estimated based on the behavioral choices made and feedback received. This allows us to measure how learning occurred during the experimental task and the subprocesses that must underlie this learning. Second-level group analyses will examine how learning in patients differs from that observed in control participants and how CR therapy alters learning efficiency and the underlying neural activity. We will correct for multiple comparisons; the voxel-level threshold will be set at P<.05 family-wise error corrected.

### Ethical Considerations

This trial has been approved by the Metro South Health Human Research and Ethics Committee (HREC/2021/QMS/67093). All protocol modifications and serious adverse events will be reported to the ethics committee. The study will be conducted in accordance with the Declaration of Helsinki and Good Clinical Practice guidelines. All participation will be based on voluntary, written, and informed consent. Participants with serious mental illness can be challenging to follow up with; thus, the researchers will make multiple attempts to contact each participant at baseline and postintervention. In cases of deterioration in mental state, the intervention will be discontinued, and the participant’s treating team will be advised. Participants will be able to withdraw at any point in the study. All data used for analysis will be deidentified.

## Results

This trial was registered in August 2018 and commenced recruiting in May 2022. As of September 2023, we have enrolled 11 healthy controls. We have also enrolled 16 individuals with SSDs, 15 into the CR group, and 1 into the TAU group. The projected completion of recruitment is September 2024. The projected final reporting date is September 2025. Results will be disseminated to mental health clinicians, researchers, and key stakeholders through peer-reviewed publications and presentations. In-kind funding is being provided by Metro South Addiction and Mental Health Services and the Translational Research Institute. This study received an Extraordinary Research Grant from the Princess Alexandra Hospital Research Foundation to assist with the purchase of fMRI scans in May 2023.

## Discussion

### Principal Findings

CR has demonstrated effectiveness in improving neurocognitive functioning in individuals with SSDs; however, the mechanisms that mediate this effect remain unclear. This study describes the unique protocol for a pre-post pilot study that aims to investigate RL as a potential mechanism for improvement following CR. In this study, we predict that at baseline, individuals with schizophrenia will exhibit deficits in RL when compared with healthy controls. Further, we predict that these deficits will be more pronounced in learning tasks that involve social versus nonsocial stimuli. This would support previous research highlighting impaired reward processing in SSDs [8], particularly in social processing [19,20]. Social reward has significant impacts on the functional outcomes of this population, and there are no current interventions that target this problem, emphasizing the importance of this area of research. Moreover, difficulty in responding to feedback has been associated with cognitive function and negative symptoms in SSDs [15-17]. Thus, we propose that by reducing RL problems, we may in turn see a reduction in negative symptoms and an improvement in cognition.

The primary focus of this study is to investigate whether RL is improved after CR in individuals with schizophrenia. Only 1 other study has specially looked at the impact of CR on reward processing in SSDs [29], and results from this research suggest CR may improve response to feedback, moderated by the severity of negative symptoms. We believe CR serves to strengthen the processes involved in rewards and motivation that are needed for participants to persevere with difficult cognitive tasks via reinforcement of learning. Learning more about the way in which CR works is important to be able to maximize the effects of the intervention. Currently, CR is undertaken for around 3 months, with 2-4 sessions per week. Understanding the mechanisms of effect may enable improvements in the programs that enable more efficient delivery of this effective intervention.

### Limitations

The lack of blinding of researchers and the nonrandomized assignment of participants to conditions are limitations of the study design. Treatment allocation is based on individuals’ preferences to minimize attrition. As the intervention group is self-selecting, arguably, these participants may be better functioning than the patient control group. However, we believe that this will not necessarily be the case. For instance, some higher-functioning participants might select to be in the control group due to time restraints (ie, because of full-time study or work) limiting their ability to complete the CR therapy sessions. Nevertheless, we aim to recruit comparable participants from the patient control group and intervention group by matching for age, sex, and premorbid function across groups. There will also be some flexibility in the delivery of CR (ie, on the web vs in person and individual vs group), depending on participants’ access to the technology to run CR sessions and their preferences. This flexible mode of delivery was aimed at minimizing attrition, which has been an issue identified in the literature, in our work, and because of group therapy shutdowns during the COVID-19 pandemic [56,57].

### Conclusions

It is hoped that the results of this study will contribute to the understanding of CR and RL in schizophrenia more generally. Greater knowledge of CR would seek to inform clinicians to develop more targeted interventions and, consequently, reduce negative symptoms and improve functional outcomes in individuals with SSDs. We hope to use this pilot to test the integrity of the protocol and plan for future funding, with the aim of progressing to a larger randomized controlled trial.

## Abbreviations

BACS: Brief Assessment of Cognition in Schizophrenia
BIS/BAS: Behavioral Inhibition System and Behavioral Activation System
BPRS: Brief Psychiatric Rating Scale
CAINS: Clinical Assessment Interview for Negative Symptoms
CDSS: Calgary Depression Scale for Schizophrenia
CIRCuiTS: Computerized Interactive Remediation of Cognition-Training for Schizophrenia
CR: cognitive remediation
fMRI: functional magnetic resonance imaging
LSP-16: Life Skills Profile
MP2RAGE: magnetization prepared 2 rapid acquisition gradient echoes
RL: reward learning
ScoRS: Schizophrenia Cognition Rating Scale
SSD: schizophrenia spectrum disorder
TAU: treatment-as-usual
TMG: Trial Management Group
TOPF: Test of Premorbid Functioning

## Data Availability

The data sets generated or analyzed during this study are not publicly available. Access to research data is restricted and governed by the Queensland Health Government.
